# Conscientiousness as a Predictor of the Gender Gap in Academic Achievement

**DOI:** 10.1007/s11162-022-09716-5

**Published:** 2022-08-16

**Authors:** Anne-Roos Verbree, Lisette Hornstra, Lientje Maas, Leoniek Wijngaards-de Meij

**Affiliations:** 1 Social and Behavioral Sciences, Utrecht University, Utrecht, The Netherlands; 2 Education, Utrecht University, Utrecht, The Netherlands; 3 Methodology and Statistics, Utrecht University, Utrecht, The Netherlands

**Keywords:** Conscientiousness, Personality, Gender gap, Academic achievement, (non-dominant) Ethnic background, Higher education

## Abstract

In recent decades, female students have been more successful in higher education than their male counterparts in the United States and other industrialized countries. A promising explanation for this gender gap are differences in personality, particularly higher levels of conscientiousness among women. Using Structural Equation Modeling on data from 4719 Dutch university students, this study examined to what extent conscientiousness can account for the gender gap in achievement. We also examined whether the role of conscientiousness in accounting for the gender gap differed for students with a non-dominant ethnic background compared to students with a dominant ethnic background. In line with our expectations, we found that conscientiousness fully mediated the gender gap in achievement, even when controlling for prior achievement in high school. This was the case among both groups of students. These findings provide insight into the mechanisms underlying the gender gap in achievement in postsecondary education settings. The current study suggests that the use of conscientiousness measures in university admission procedures may disadvantage male students. Instead, the use of such measures may be a fruitful way to identify those students who may benefit from interventions to improve their conscientiousness. Future research could examine how conscientiousness can be fostered among students who are low in conscientiousness.

In recent decades, female students have been more successful in higher education than their male counterparts in the United States and other industrialized countries (e.g., Buchmann & DiPrete, 2006). A higher percentage of women enrolls in higher education, female students obtain higher grades and earn more credits than male students, and are more likely to graduate (Conger & Long, 2010). Nevertheless, there is no consensus regarding why female students outperform male students in postsecondary education (Sax & Harper, 2007).

Since general cognitive ability does not differ by gender^1^ (Carvalho, 2016), other explanations for the gender gap have been proposed which focus on economic factors or individual differences between men and women. A promising explanation of the latter kind is differences in personality, particularly in conscientiousness. Conscientiousness, which includes being hardworking, reliable, organized, ambitious, self-disciplined, and persevering (McCrae & Costa, 1987), is the personality trait that most strongly predicts academic achievement (e.g., Trapmann et al., 2007). As several studies found that women are higher in conscientiousness than men (e.g., Mac Giolla & Kajonius, 2018; Nguyen et al., 2005), this trait may explain the relation between gender and achievement.

In the current study, we examine the gender gap and the role of conscientiousness in explaining this gap separately for students with a dominant and students with non-dominant ethnic background. This study is conducted in the Dutch context and defines students with a non-dominant ethnic background as students with a non-Western ethnic background (i.e., students who were born or with at least one parent born in a non-Western country^2^), aligning with the definition of Statistics Netherlands (2018). The gender gap in academic achievement is observed both among students with a dominant ethnic background and students with non-dominant ethnic background (e.g., Fleischmann & Kristen, 2014). However, the role conscientiousness plays in accounting for the gender gap might differ between these groups of students. Research has shown that gender differences in personality are larger in countries with more gender-equal, Western cultures (e.g., Mac Giolla & Kajonius, 2018). On the one hand, this may suggest that conscientiousness explains the gender gap better for students with a dominant ethnic background than for students with a non-dominant (i.e., non-Western) ethnic background. On the other hand, it can be hypothesized that there is a stronger relation between conscientiousness and achievement for students with a non-dominant ethnic background as these students are faced with more stressors and challenges in college (e.g., Wei et al., 2011). Conscientiousness may serve as a protective factor to overcome these stressors (e.g., Bartley & Roesch, 2011).

The present study examines to what extent conscientiousness explains the gender gap in academic achievement in postsecondary education among students with a dominant and non-dominant ethnic background. Thereby, the current study aims to gain insight into the mechanisms underlying the gender gap and how these may differ for students with different ethnic backgrounds.

## Literature Review

The following section describes prior literature relevant to the gender gap in achievement in higher education. We present an overview of the emergence and origins of the gender gap in higher education, the potential explaining role of conscientiousness, and how the role of conscientiousness in explaining the gender gap may differ for students with a non-dominant ethnic background.

## Gender Gap in Achievement in Higher Education

In recent decades, women have been more successful in postsecondary education than men in the United States and in Europe (e.g., Buchmann & DiPrete, 2006; McDaniel, 2013). Both female college enrollment rates (e.g., Francesoni & Parey, 2018; UNESCO, 2012) and graduation rates (e.g., Conger & Long, 2010; McDaniel, 2013) are higher than those of male students and female students obtain more credits and better grades than their male counterparts (e.g., Conger & Long, 2008, 2010). This gender gap in enrollment and academic achievement has been observed among different ethnic groups (Conger & Dickson, 2017) and for all types of educational institutions (e.g., public, private; Goldin et al., 2006).

In contrast, from the post-World War II period to the 1980s, there was a gender gap in enrollment and graduation rates favoring men (Goldin et al., 2006). During this period, female enrollment and graduation rates increased (UNESCO, 2012) resulting in gender parity in postsecondary education (Ewert, 2012). Eventually, the gender gap reversed with women being more successful in higher education in the United States and elsewhere. While the traditional gender gap favoring men has been investigated extensively, only recently more attention has been paid to the gender gap in favor of women.

Research into explanations for this gender gap has two major foci: economic factors and individual differences between men and women (Doherty et al., 2016). The first explanation regards the gender gap in higher education as a result of the changed and changing economic position of women (e.g., Goldin et al., 2006; Vincent-Lancrin, 2008), which has led to an increase in women’s expected economic returns of going to college. In turn, this resulted in increased female enrollment and graduation rates (Goldin et al., 2006). Yet, this is likely only part of the explanation (DiPrete & Buchmann, 2006; Ewert, 2012) and in an age of (more) equalized opportunities, individual differences may have more importance in accounting for the gender gap (Xu & Wu, 2015).

Prior research has demonstrated that many individual factors predict college enrollment and achievement. Many of these factors start shaping an individual’s educational career before being aware of the gross characteristics of labor or marriage markets (DiPrete & Buchmann, 2006). Therefore, research into potential explanations for the gender gap has focused on gender differences in biological traits or developmental trajectories, in behavioral and social factors, and gender differences resulting from the socialization process of gender norms (Xu & Wu, 2015). In particular, noncognitive characteristics, such as the ability to follow directions, work in groups, and organize materials (Jacob, 2002), are assumed to have enabled women to thrive, which may have contributed to the gender gap in academic achievement in favor of women in Western countries.

Gender differences in personality may explain the gender gap in achievement as personality is an important predictor of postsecondary academic achievement. O’Connor and Paunonen (2007) outline three justifications for this relation. First, personality traits are predictive of certain behavioral tendencies (e.g., perseverance, talkativeness; Rothstein et al., 1994) that can influence academic success. Compared to men, women’s personalities may lead to behavioral tendencies that are more beneficial for academic success. Second, while cognitive measures indicate what a student *can* do, measures of personality traits may have additional predictive value for academic achievement because they provide information on what a student *will* do (Furnham & Chamorro-Premuzic, 2004). Thus, while men and women have a similar level of cognitive ability (Carvalho, 2016), gender differences in personality may result in differences between what male and female students do in favor of their academic achievement. The third argument for personality traits serving as predictors of academic performance is that cognitive ability might lose its predictive power at the level of higher education (e.g., Furnham et al., 2003). Research has shown that the relation between cognitive ability and academic performance is often weaker among university students compared to elementary and secondary school students (O’Connor & Paunonen, 2007). This may be the result of possible barriers to entering higher education being overcome by college students and of range restriction in their intelligence. In turn, this suggests that personality traits, and thus differences by gender, are particularly predictive for achievement in university settings (Furnham et al., 2003).

A fourth potential but controversial reason for why personality might predict academic achievement is that women’s personalities may be better aligned with the (higher) education system than those of men (Almås et al., 2016). For example, teachers may favor behaviors that are more typical for women (Vincent-Lancrin, 2008). Nevertheless, opinions and the literature are ambiguous about whether this serves as a reason for personality predicting achievement, and more specifically as an explanation for the gender gap in education.

## Conscientiousness

Conscientiousness is one of the personality dimensions of the Five-Factor Model (FFM), also called the Big Five. The FFM has received considerable support in terms of its robustness and generalization across theoretical frameworks, cultures, and assessment methods (Hogan & Ones, 1997). Conscientiousness, also referred to as dependability, conformity, or will to achieve (Nguyen et al., 2005; Poropat, 2009), entails being careful, thorough, responsible, organized, achievement-oriented, diligent, self-disciplined, and persevering (John & Srivastava, 1999; Nguyen et al., 2005; see Costa & McCrae, 1995, for information on its facets).

Multiple studies have found that conscientiousness strongly predicts academic achievement and in meta-analytic investigations of personality and academic achievement, conscientiousness emerges as the strongest and most consistent personality trait predicting (postsecondary) academic achievement (O’Connor & Paunonen, 2007; Poropat, 2009; Trapmann et al., 2007). It predicts achievement with the same size as and beyond intelligence (Poropat, 2009). Because of the incremental validity of conscientiousness as a predictor of academic achievement beyond cognitive measures, researchers have even suggested using the assessment of conscientiousness for student guidance (e.g., Lievens et al., 2002) and admission and selection processes in higher education (e.g., Conard, 2006; Furnham et al., 2003; Kappe & Van der Flier, 2012; Kling et al., 2012).

It has been proposed that personality traits do not impact academic achievement directly, but through proximal constructs that are more closely related to behavior (Chen et al., 2000). Specifically, the relation between conscientiousness and achievement is mediated by motivation (Hazrati-Viari et al., 2012; Richardson & Abraham, 2009) and its resulting proximal behaviors (e.g., increased class attendance; Conard, 2006; more time spent on the task at hand; Biderman et al., 2008). Other proximal behaviors resulting from conscientiousness that are important to achievement are, for example, being organized, diligent, and hardworking (O’Connor & Paunonen, 2007) and employing effective learning strategies (Komarraju et al., 2011).

Various studies found that women are somewhat higher in conscientiousness than men (e.g., Keiser et al., 2016; Kling et al., 2012; Mac Giolla & Kajonius, 2018; Nguyen et al., 2005; Vianello et al., 2013). Gender differences have also been found for the other Big Five traits (e.g., Mac Giolla & Kajonius, 2018; Vianello et al., 2013). Two theoretical accounts, biological and social-psychological, might explain gender differences in personality (Costa et al., 2001; Vianello et al., 2013). The biological account ascribes this phenomenon to innate sex differences, while the social-psychological account states that gender differences derive from social expectations of how men and women should think, feel, and behave (i.e., the social role model; Eagly, 1987). These gender roles are internalized early in life through socialization processes and shape personality traits and their proximal behaviors later in life (Vianello et al., 2013).

To summarize, since men and women have been found to differ in conscientiousness (Costa et al., 2001) and since conscientiousness predicts academic achievement, conscientiousness may account for the gender difference in higher education achievement. Some studies examined similar questions. Carvalho (2016) investigated whether certain personality dimensions mediated the gender-achievement relation among adolescents and found that disconstraint (i.e., difficulties with self-control and norm compliance, tendencies toward impulsive action) mediated this relation due to a lower level of disconstraint in young women. In a similar vein, several studies examined the phenomenon that, compared to male students, female students earn higher college grades than expected based on their admission test scores. Research into this so-called *underprediction* of female performance in U.S. colleges has shown that conscientiousness explains female students’ higher than expected achievement (Kling et al., 2012; cf. Keiser et al., 2016). Thus, conscientiousness mediated the relation between gender and the underprediction of performance. This shows that women’s higher level of conscientiousness led to higher grades than expected based on their admission scores, which is likely to be a result of proximal behaviors (e.g., working hard) that follow from being conscientious. Based on these studies, it seems likely that conscientiousness serves as a mediator of the gender gap in academic achievement. Since this has not been investigated directly, this will be done in the present study.

## The Gender Gap Among Students with a Non-Dominant Ethnic Background

Few studies looked at the presence of the gender gap in achievement among students with different ethnic backgrounds (Fleischmann & Kristen, 2014; Steele-Johnson & Leas, 2013). Similar to findings among the general higher education population, those studies consistently found a gender gap in favor of female students among students with a non-dominant ethnic background (e.g., Conger & Dickson, 2017; Conger & Long, 2008; Fleischmann & Kristen, 2014; Schippers et al., 2015; Steele-Johnson & Leas, 2013). Nevertheless, it is unclear whether the size of the gender gap is similar for students with a dominant and non-dominant ethnic background. Also, it is unclear whether the factors explaining the gender gap may differ for these groups of students (Davis & Otto, 2016), for example due to cultural differences.

There is evidence that gender differences in personality traits are larger in countries with higher levels of human development, which entails longer and healthier lives, equal access to education, economic wealth, and egalitarian cultures in which women’s opportunities are more equal to those of men (Costa et al., 2001; Mac Giolla & Kajonius, 2018; Schmitt et al., 2008). This means that gender differences in personality tend to be larger in Western countries, compared to non-Western countries. However, it is uncertain whether this variability in gender differences in personality between countries (Costa et al., 2001) is resembled in differences between groups with a dominant and non-dominant ethnic background in Western countries (cf. Khoudja & Fleischmann, 2015). There might be a smaller gender difference in personality among students with a (non-Western) non-dominant ethnic background because immigrant families may (partly) maintain the attitudes from their (non-Western) culture of origin (e.g., Fleischmann & Kristen, 2014). This would imply a weaker relation between gender and conscientiousness among students with a non-dominant ethnic background than among other students, and means conscientiousness would account to a lesser extent for the gender gap among students with a non-dominant ethnic background than among students with a dominant ethnic background.

However, it could also be hypothesized that conscientiousness plays a stronger role in explaining achievement among students with a non-dominant background than students with a dominant ethnic background, in which case it may also be a stronger predictor of the gender gap among students with a non-dominant ethnic background. That is, even though most students find university life somewhat challenging, students with a non-dominant ethnic background can experience additional stressors in higher education due to their minority status (e.g., Alvarez et al., 2009; Carter et al., 2013; Wei et al., 2011) which may negatively impact achievement (Buddington, 2002; Greer & Chwalisz, 2007). Conscientiousness has been found to serve as a protective factor from stress due to the selection of appropriate coping strategies (Bartley & Roesch, 2011; Campbell-Sills et al., 2006) and aspects such as maintaining task commitment and persisting when encountering challenges (Kwok et al., 2007). Moreover, the tendency of conscientious individuals to plan and prioritize may reduce the number of stressors encountered (Besser & Shackelford, 2007; Connor-Smith & Flachsbart, 2007). In short, students with a non-dominant ethnic background are faced with more stressors in higher education which can hamper their academic achievement. As conscientiousness serves as a protective factor against this, there may be a stronger relation between conscientiousness and achievement for students with a non-dominant ethnic background than for students with a dominant ethnic background. In that case, gender differences in conscientiousness among students with a non-dominant ethnic background may have stronger effects on achievement than for students without a dominant background.

The impact of ethnic background on gender differences in conscientiousness and on the conscientiousness-achievement relation has only scarcely been examined. To our knowledge, only one study (Steele-Johnson & Leas, 2013) has researched whether ethnicity affected the relation between personality and academic achievement. They found that the effect of conscientiousness on academic achievement was similar among U.S. college students with different ethnic backgrounds. It is not clear whether these findings are robust and generalizable to the European context. Therefore, the present study will take ethnic background into account when examining the role of conscientiousness in explaining the gender gap in higher education. Due to the mixed directions previous research points to, we did not formulate a hypothesis about the effect of ethnic background on the role of conscientiousness in accounting for the gender gap in achievement.

## Present Study

Relatively little research has investigated why men fall behind in postsecondary education (Conger & Long, 2010). This leaves the mechanisms that affect differences in male and female students’ achievement unexplored (Jacob, 2002; Nguyen et al., 2005). Therefore, the present study examines the role of conscientiousness in the relation between gender and academic achievement. This is of additional importance in the context of various current issues. First, the COVID-19 pandemic required higher education to switch from physical to a (partly) online form. Several aspects of conscientiousness such as motivation and self-discipline are especially important in online education (e.g., Gorbunovs et al., 2016; Waschull, 2005). Therefore, the gender gap in achievement may be enlarged due to COVID-19. Second, recent attention to inclusion and equal opportunities in higher education in both research and the media (e.g., Collins et al., 2019) further illustrates the relevance of understanding differences in attainment based on gender and ethnic background. Moreover, there is an increased interest in the U.S. and Europe in the use of noncognitive criteria, such as students’ scores on personality tests, for admission to higher education (Hossler et al., 2019; Niessen & Meijer, 2017). If conscientiousness accounts for the gender gap in academic achievement, including this trait in admission procedures may disadvantage male students. Instead, since personality traits, including conscientiousness, are malleable (e.g., Roberts et al., 2017), higher education institutions may consider promoting conscientiousness in students who are low on this trait. Teaching students to be more organized, careful, and to think about the consequences of their actions can result in students becoming more conscientious (Roberts et al., 2017).

**Fig. 1:**
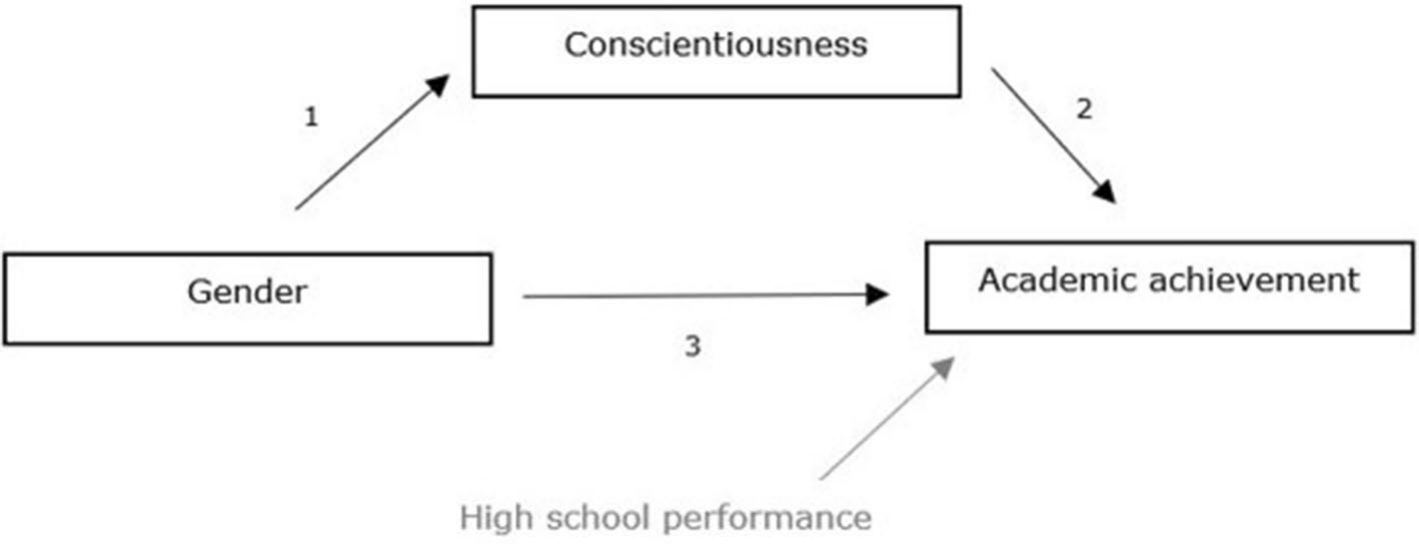
Conceptual model of conscientiousness mediating the gender-achievement relation

To conclude, it is necessary to understand the role of conscientiousness in the gender gap in achievement and how this differs for students with different ethnic backgrounds. This is not only important in theoretical respect but also to inform university administrators about admission decisions and universities about providing support services to increase students’ chances of academic success (Sheard, 2009). To address these gaps in the current knowledge, the present study investigates to what extent conscientiousness explains the gender gap in academic achievement in higher education over and above prior performance in high school (Fig. 1). In addition, we examine whether the potential mediating role of conscientiousness differs for students with a dominant and non-dominant ethnic background. For both groups, we expect conscientiousness to serve as a mediator in the relation between gender and achievement. For students with a non-dominant ethnic background, conscientiousness could either account to a lesser extent for the gender gap due to a weaker relation between gender and conscientiousness (Fig. 1, relation 1) or to a stronger extent due to a stronger relation between conscientiousness and academic achievement (Fig. 1, relation 2).

Although our study was conducted in the Dutch context, our study is also relevant for other countries as the gender gap in favor of women is observed in most Western countries. Also, students with a non-dominant ethnic background have substantial educational disadvantages in both the Netherlands and other European countries and similar patterns have been observed for racial minorities in the U.S. (e.g., Heath et al., 2008), including African-American and Latino students. Further, in the current study, we used two indicators of first-year academic achievement to investigate the gender gap *during* enrollment rather than looking at enrollment or graduation rates, which may mask achievement differences among students.

## Method

The present study had a quantitative longitudinal/prospective design in which questionnaire data were collected from a large sample of (prospective) university students prior to enrollment. The questionnaire was part of a compulsory program just before enrollment in which prospective students are required to participate. Student achievement data were collected during their first year of enrollment in university.

### Participants

Participants were 4719 Dutch students (55.7% women; *M*_age_ = 19.18 years, *SD* = 2.69) who enrolled in a nonselective bachelor program (i.e., without selection process prior to enrollment) at Utrecht University in 2015. Of the participants, 358 students had a non-dominant ethnic background. Aligning with the definition put forward by Statistics Netherlands (2018), students with a (non-Western) non-dominant ethnic background are defined as students who were born (first-generation) or with at least one parent born (second-generation) in a non-Western country (i.e., countries in Africa, Asia [including Turkey, excluding Japan, Indonesia], and Latin America]). Participants were enrolled in the following faculties: Faculty of Geosciences (9.9%), Humanities (26.7%), Law, Economics, & Governance (23.9%), Science (17.9%), Social & Behavioral Sciences (21.7%). In the Netherlands, the student population in higher education is rather mixed, although female students and Dutch students (i.e., without a migration background) are somewhat overrepresented (Youth Monitor, 2021).

### Procedure

Data were collected during the spring of 2015 as part of a compulsory matching program that took place 2–3 months prior to enrollment. In the Netherlands, universities are obliged to offer some form of matching program (e.g., interviews, questionnaires, lectures on campus) to prospective students (Van der Wende, 2012), which consists of activities to help students assess whether their chosen program matches their interests, capabilities, and study skills. The aim of these programs is to decrease dropout during the first year of enrollment.^3^

The matching program in which the participants of this study participated included filling out an online questionnaire on motivation, personality, and expectations about the bachelor program students planned to enroll in. This questionnaire differed per bachelor program, but for all programs, it included the same conscientiousness measure. Prospective students also came to the university for a day during which they attended a lecture, participated in a tutorial, and took a practice exam to familiarize them with their preferred program. At a later stage, students were asked for passive consent for linking the enrollment data to data from university registers and for the use of this data for research ends, which gave students the opportunity to opt-out of the study. For students who gave consent, data about their demographic characteristics (i.e., gender, ethnic background), high school performance, and academic achievement during the first year of enrollment were obtained from university registers, linked to students’ answers on the matching questionnaire, and anonymized. Ethical approval for this study has been granted by the local Institutional Review Board.

### Measures

#### Conscientiousness

Students’ self-perceived conscientiousness was measured with nine items (see Appendix 2) that came from the Dutch translation of the Big Five Inventory (Denissen et al., 2008). The items were answered on a 5-point Likert scale ranging from (1) *strongly disagree* to (5) *strongly agree*. Four items were recoded so that higher scores reflected being more conscientious. Based on a reliability analysis, one item was deleted, resulting in Cronbach’s alpha = .81. This implies very good internal consistency of the scale in this sample (Kline, 2011). The eight items were used as indicators of the latent construct conscientiousness.

#### Achievement and Prior Performance in High School

While most studies employ GPA as a single indicator of academic achievement, we used an additional indicator of academic achievement to better represent its multidimensional nature (O’Connor & Paunonen, 2007). Besides average grade (on a scale from 1 to 10), we included the number of credits that students obtained during their first year of enrollment. These credits are based on the European Credit Transfer System (ECTS; see e.g., Schippers et al., 2015 for details) and are awarded when students pass a course. They represent the number of courses students successfully completed during their first year. Both average grade and credits were used as indicators of the latent construct academic achievement. Students’ average high school performance (on a scale from 1 to 10) was used as indicator of prior achievement. We controlled for prior achievement, which is likely to be the result of a combination of cognitive, social, and dispositional variables (Richardson & Abraham, 2009) and has consistently been found to be a strong predictor of higher education academic achievement (Conger & Long, 2008; Sax & Harper, 2007). Therefore, controlling for high school performance enables us to rule out the impact of such other variables on academic achievement and to determine whether, at similar levels of prior performance, gender differences in academic achievement are present and can be explained by conscientiousness. We excluded prior achievement for students older than 25 at enrollment (*n* = 12) due to a lack of comparability to the prior achievement of younger students and a potential lower predictive value of this variable for these students.

### Data Analysis

The hypothesized model was tested using Structural Equation Modeling (SEM) in Mplus, version 8.3 (Muthén & Muthén, 2019). SEM can be used to model complex relations between multiple variables, including both latent variables, which cannot be measured directly (e.g., conscientiousness), and observed variables (Geiser, 2013; Teo et al., 2013). In addition, SEM corrects for measurement error in the indicators (e.g., items) that are used (Geiser, 2013; Teo et al., 2013). Full information maximum likelihood estimation was used to handle missing data, which ranged from 7.5 to 16.1% for all variables except for gender (0%).

First, the hypothesized model (Fig. 1) was tested for all students to examine the relations without taking ethnicity into account, as is often done in existing research. Then, measurement invariance (i.e., *un*biasedness of tests; Wicherts & Dolan, 2010) by ethnic background for the latent variables conscientiousness and achievement was established for a two-factor confirmatory factor analysis. Using a stepwise approach in which equality constraints were added (equal factor loadings, both equal factor loadings and equal intercepts, respectively), it was examined whether measurement invariance was tenable (cf. Wicherts & Dolan, 2010). Next, by using multigroup analysis, the structural model was specified for students with a dominant and non-dominant ethnic background separately. Based on these models, it was examined whether the structural parameter estimates could be constrained to be equal for both groups of students.

To assess goodness-of-fit, we used the chi-square statistic (*p* > .05 indicating good fit). However, as this statistic is sensitive to sample size (Marsh et al., 1988), we primarily looked at other goodness-of-fit indices (Schermelleh-Engel et al., 2003): Root Mean Square Error of Approximation (RMSEA; .06–.08 indicating adequate fit, ≤ .05 indicating good fit), Comparative Fit Index (CFI; ≥ .95 indicating acceptable fit, ≥ .97 indicating good fit), Tucker-Lewis Index (TLI; see CFI for cut-off values), and Standardized Root Mean Square Residual (SRMR; .05–.08 indicating acceptable fit, < .05 indicating good fit). We used these indices also to compare nested models (i.e., to examine whether equality constraints lead to decreased model fit) in addition to the chi-square difference test due to its sensitivity to sample size. Modification indices (MIs) were considered, if theoretically sensible (Kline, 2011), in case of poor model fit. Mediation was assessed by 95% bias-corrected bootstrap confidence intervals (BC CIs), based on 10,000 bootstrap samples, for the indirect effects.

Before conducting the analyses, multivariate normality and independence of exogenous variables (i.e., gender and high school performance) were checked. Due to some violations of normality, maximum likelihood estimation with robust standard errors (MLR) was used. This is the only estimator in Mplus that yields a chi-square statistic that is robust to nonnormality *and* is available with missing data (Muthén & Muthén, 2010). The independence of exogenous variables was met.

## Results

First, descriptive statistics of the included variables are presented. Next, the results of the analyses investigating conscientiousness as mediator of the gender gap in achievement are described. Finally, results of the analyses investigating whether the mediation model differs for students with a dominant and non-dominant ethnic background are presented.

### Descriptive Statistics

Table 1 presents descriptive statistics for conscientiousness, the indicators of academic achievement, and high school performance. Table 2 presents correlations between gender, ethnic background, high school performance, and the latent variables conscientiousness and academic achievement.

**Table 1:** Mean (SD) conscientiousness, academic achievement, and high school performance

	Students with a dominant ethnic background		Students with a non-dominant ethnic background		Total	
	Males	Females	Males	Females	Males	Females
Conscientiousness^a^	3.58 (0.53)	3.87 (0.52)	3.64 (0.66)	3.92 (0.50)	3.58 (0.55)	3.88 (0.52)
GPA	6.87 (0.63)	6.98 (0.61)	6.78 (0.57)	6.83 (0.57)	6.85 (0.62)	6.97 (0.60)
Credits	49.16 (18.21)	53.05 (15.53)	47.22 (18.07)	48.84 (17.98)	48.79 (18.16)	52.62 (15.99)
High school performance	6.71 (0.56)	6.83 (0.54)	6.55 (0.49)	6.69 (0.52)	6.69 (0.55)	6.81 (0.54)
Range of *n*^b^	1455–1606	1766–1987	113–131	122–169	1720–1898	2173–2467

^a^Conscientiousness indicates the mean of the eight conscientiousness items

^b^Range of *n* indicates per column the *n* on which the presented means and standard deviations are based, *n* varied due to missing data

**Table 2:** Correlations between ethnic background, gender, conscientiousness, academic achievement, and high school performance

	1	2	3	4	5
1 Ethnic background	–				
2 Gender (ref. = female)	− .01	–			
3 Conscientiousness	.03	− .30*	–		
4 Academic achievement	− .07*	− .13*	.29*	–	
5 High school performance	− .07*	− .11*	.23*	.72*	–

*p < .001

### Conscientiousness as Mediator of the Gender Gap

**Fig. 2:**
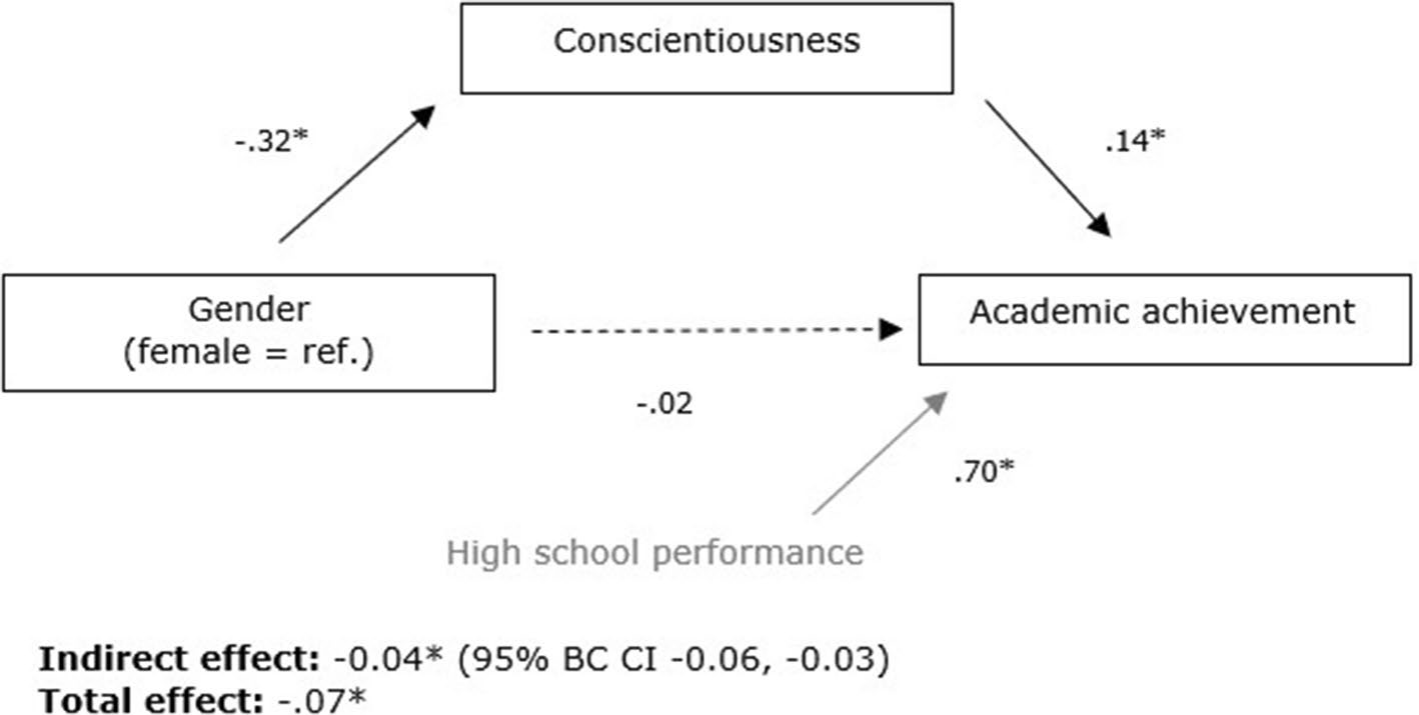
Standardized parameter estimates of the SEM of conscientiousness mediating the gender-achievement relation for all students, *p < .001

First, we examined whether conscientiousness mediated the relation between gender and achievement for all students together. This did not directly result in adequate model fit. Accordingly, based on MIs and theoretical grounds, two correlations were added between the error terms of two pairs of indicators of the latent variable conscientiousness. The modified model fit was acceptable, χ^2^(49) = 814.40, *p* < .001, RMSEA = .06, CFI = .93, TLI = 0.90, SRMR = .06. The factor loading of each indicator to its latent factor was high and significant (standardized factor loadings ranged from .50 to .90, all *p* < .001). Figure 2 presents the standardized parameter estimates of this model. All structural parameters were significant, except for the direct effect of gender on achievement. The direction of the relations was in line with our hypotheses: male students reported lower conscientiousness and conscientiousness was positively linked to academic achievement. According to the guidelines of Cohen (1988), the corresponding effect sizes can be interpreted as small-to-medium and small, respectively. While the direct effect of gender on achievement was nonsignificant, both the total effect and the indirect effect of gender on achievement were significant. In line with our hypotheses, this indicates the presence of complete mediation of the relation between gender and achievement through conscientiousness. The model explained 10.4% of the variance in conscientiousness and 52.0% in achievement. The amount of explained variance in achievement was mostly due to high school performance. Removal of this variable from the model resulted in 10.4% explained variance in achievement.

### Differences by Ethnic Background

Before examining whether the mediation model differed for students with a dominant and non-dominant ethnic background, strong measurement invariance (equal factor loadings and equal intercepts) across groups of the measurement model was established for a model with the (correlated) latent variables conscientiousness and achievement (see Appendix 1). Then, we could test the structural mediation model. The full model without equality constraints on the structural parameters across the groups (i.e., the relations between the included concepts were not restricted to be equal across groups) had an acceptable model fit, χ^2^(115) = 793.30, *p* =  < .001, RMSEA = .06, CFI = .93, TLI = 0.92, SRMR = .07.

Next, to investigate whether conscientiousness plays a different role in explaining the gender gap for students with a dominant and non-dominant ethnic background, we tested whether the direct paths (except for the path from high school performance to achievement) and indirect path could be constrained to be equal for both groups. This model still showed an acceptable fit, χ^2^(118) = 793.35, *p* =  < .001, RMSEA = .06, CFI = .93, TLI = 0.92, SRMR = .07. Also, the chi-square difference test was nonsignificant, χ^2^_diff_(3) = 2.47, *p* = .482. Hence, the mediation model could be considered to be similar for both groups of students.

**Fig. 3:**
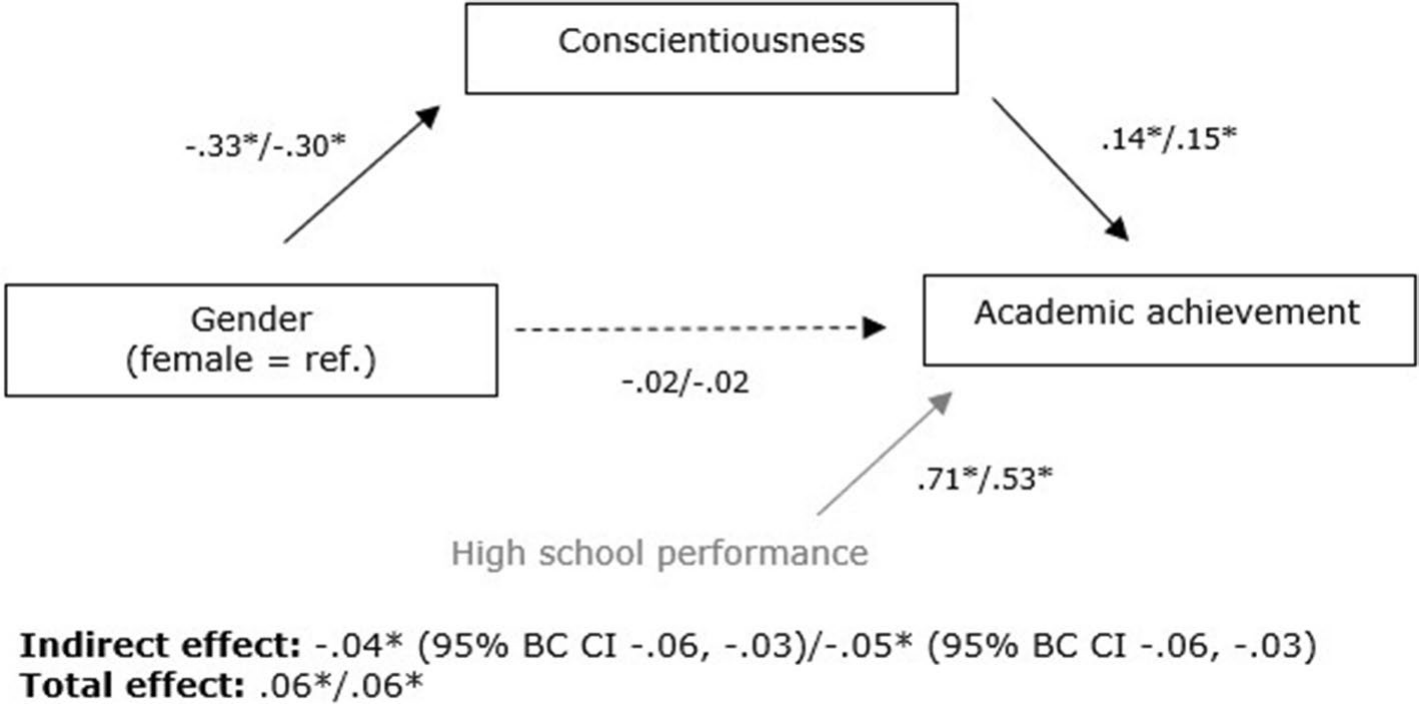
Standardized parameter estimates by ethnicity of the SEM of conscientiousness mediating the gender-achievement relation (left estimates of students with a dominant ethnic background, right estimates of students with a non-dominant ethnic background which differ due to differences in variances; the estimates of the indirect effect are not significantly different), *p < .001

Figure 3 shows the standardized parameter estimates for this final model (see Appendix 2 for the standardized factor loadings). The findings indicate that for both groups of students, gender was related to conscientiousness, with men reporting lower levels of conscientiousness, and conscientiousness positively predicted academic achievement. The corresponding effect sizes can be interpreted as a small-to-medium and a small relation, respectively. Also, both the total effect of gender on academic achievement and the indirect effect through conscientiousness were significant. These results suggest that, as expected, conscientiousness mediated the gender-achievement relation for both groups. As the direct effect was not significant, this suggests that the mediation is complete. Full mediation was found even when controlling for high school performance. In the final model, conscientiousness, together with high school performance, explained 53.5% and 33.2% of the variance in academic achievement for students with a dominant and non-dominant ethnic background, respectively. The explained variance was mostly due to high school performance. Removal of this variable resulted in 11.1% (students without a dominant ethnic background) and 12.5% (students with a non-dominant ethnic background) explained variance in achievement. Gender explained 10.7% and 8.8% of the variance in conscientiousness for students with a dominant and non-dominant ethnic background, respectively.

## Discussion

In recent decades, a gender gap in higher education is observed with female students having higher academic achievement than their male counterparts in Western countries. The present study examined to what extent the personality trait conscientiousness can explain the gender gap in academic achievement and whether the role of conscientiousness in explaining this gap differed between students with a dominant and non-dominant ethnic background. The findings of the current study indicate that, for both groups of students, conscientiousness explained the gender gap in academic achievement over and above prior performance in high school. Thereby, the present study yielded more insight into the origins of the gender gap. In addition, we extended existing research by exploring differences by ethnic background.

### Key Findings and Explanations

We found complete mediation of the gender gap in academic achievement by conscientiousness, with a small-to-medium effect size. However, although we found complete mediation, we cannot exclude that other factors may also contribute to the gender gap in academic achievement. In fact, since the test of the direct effect is less powerful than that of the indirect effect (Kenny & Judd, 2014), statistical results alone are insufficient to claim complete mediation. Possibly, other noncognitive student characteristics also contribute to the gender gap in achievement. For example, women have higher levels of skills that are increasingly emphasized in higher education, such as teamwork and communication (Karim et al., 2012). This is an interesting avenue for future research. Nevertheless, even though other factors may play a role, the findings of our study still suggest that conscientiousness is an important factor in explaining the gender gap in academic achievement in postsecondary education that is observed in industrialized countries. This finding is in line with previous research that found that women are higher in conscientiousness than men (e.g., Kling et al., 2012; Nguyen et al., 2005; Vianello et al., 2013) and that conscientiousness is predictive of academic achievement (e.g., O’Connor & Paunonen, 2007; Poropat, 2009; Trapmann et al., 2007). Conscientiousness explained the gender gap in academic achievement even when accounting for high school achievement, indicating that personality has predictive utility over and above prior performance.

Our finding of the importance of conscientiousness in explaining the gender gap may be especially relevant since the COVID-19 pandemic has led to an increase in online and hybrid teaching in which students have to be more independent and disciplined, which may favor students who are more conscientious. Also, this finding raises the question of which factors account for gender differences in conscientiousness and which factors explain the relation between conscientiousness and achievement. Empirical attention to what accounts for both examined relations is needed (cf. O’Connor & Paunonen, 2007). Regardless of the underlying factors, the findings of this study show that conscientiousness is important in accounting for the gender gap in academic achievement.

Moreover, we did not find differences between students with a dominant and non-dominant ethnic background concerning the gender gap in achievement and the role of conscientiousness in explaining the gender gap. This implies that the female advantage prevalent among students with a dominant ethnic background generalizes to first- and second-generation students with a non-dominant ethnic background. A potential explanation is that the similar-sized gender gap is due to the influence of educational institutions and peers on the educational achievement of students with a non-dominant ethnic background (Fleischmann & Kristen, 2014). More research is needed into the specific mechanisms that operate in the patterns of gendered educational outcomes among students with a non-dominant ethnic background.

In addition, the relations between gender and conscientiousness and between conscientiousness and academic achievement did not differ between students with a dominant and non-dominant ethnic background. The latter finding is similar to that of Steele-Johnson and Leas (2013) who did not find a difference in the effect of conscientiousness on academic achievement between students with different ethnic backgrounds. The investigation in the current study builds on research that shows that gender differences in personality vary across countries. It is currently not clear whether this variation transfers to ethnic groups within a single country, which points to the need for future research. In addition, the scarcity of research into potential ethnic differences in the relation between conscientiousness and achievement points to the need to investigate this further. This will advance a knowledge base for understanding conscientiousness among different groups of students.

### Limitations and Suggestions for Future Research

The present study has some limitations. We used a dichotomy between students with a dominant ethnic background and with a non-dominant ethnic background due to having an insufficient number of students with a non-dominant ethnic background to make further distinctions between different ethnic groups. There may be differences between different ethnic groups in the gender gap in academic achievement and in the role of conscientiousness in explaining the gender gap. Nevertheless, higher education participation is lower among all groups with a (non-Western) non-dominant ethnic background, with the gender gap present among these groups (e.g., Youth Monitor, 2021). In addition, the definition of persons with a non-Western ethnic background is based on cultural similarities between these groups and differences with the Dutch population (Helberg-Proctor et al., 2017). Still, future research, for example based on national data, could distinguish between different ethnic groups when examining the gender gap and the role of conscientiousness. Furthermore, research in other countries is needed, including those with different populations, as the results of the current study are potentially not generalizable to those populations.

In addition, while we included important predictors (i.e., prior performance in high school, conscientiousness) of academic achievement in our study, other variables with predictive value for achievement, such as students’ self-efficacy, class attendance, time spent studying, and social interaction in courses (cf. Schneider & Preckel, 2017), were not included. Future research could include additional predictors of academic achievement in higher education to examine to what extent personality explains the gender gap in academic achievement over and above other predictors.

It is important to note that gender differences in personality are small relative to individual variation within genders (Costa et al., 2001). Besides, a self-presentation effect may occur when using self-report measures so that these measures partly reflect social norms and self-expectations about gender roles. This is illustrated by a study that found smaller gender differences in personality on implicit measures of the Big Five traits than on explicit measures (Vianello et al., 2013). Moreover, dichotomization by gender has been criticized (e.g., Vantieghem et al., 2014). However, the goal of our study is not to put men and women at two ends of the spectrum without recognizing their overlapping characteristics. Nevertheless, there is an actual gender gap in academic achievement in higher education that can at least in part be explained by conscientiousness. Yet, more insight is needed into the factors explaining the gender gap, and thereby into factors that may help to resolve the gender gap.

Furthermore, the gender gap in academic achievement, as well as the role of conscientiousness in accounting for the gender gap, may differ by academic major. A recent study found no differences between majors in the predictive value of conscientiousness for achievement while controlling for gender (Verbree et al., 2021). Future research could further examine gender gaps in academic achievement by academic major and whether the role of conscientiousness in accounting for the gender gaps differs for different academic majors.

### Implications

The present study has important implications for higher education. First, the findings indicate that women tend to score higher on conscientiousness than men. As such, using conscientiousness measures in admission and selection processes will likely disadvantage male students. Nevertheless, there has recently been increased interest in the U.S. and Europe in using noncognitive measures such as conscientiousness measures in addition to achievement measures for student selection (Niessen & Meijer, 2017) and various authors have suggested using conscientiousness for admission and selection processes in higher education (e.g., Conard, 2006; Furnham et al., 2003; Kappe & Van der Flier, 2012; Kling et al., 2012). Hence, the findings of the present study suggest it is important for educators to be aware that using conscientiousness for admission purposes could result in a lower male enrollment rate. Instead, conscientiousness measures administered to prospective students may be used to detect which students need support. That is, even though including conscientiousness measures in selection procedures may be problematic, our results do indicate that conscientiousness is a valuable trait for students that helps them to succeed in higher education. Using a conscientiousness measure in admission procedures can help to detect which students may be at risk for failure due to low conscientiousness and who may benefit from additional services or interventions to foster conscientiousness.

Research on interventions has shown that personality traits can be altered (Magidson et al., 2014). Applying such interventions to foster (prospective) students’ conscientiousness may help students, in particular male students, to succeed in higher education. For example, students can be taught certain behaviors such as being more organized and careful in their work, can be encouraged to think about the consequences of their actions, and can be helped in increasing their self-control. Roberts et al. (2017) suggest that eventually, if such changes in behavioral states become extended, internalized, and automatic, these changes in states will infer personality trait change, that is, becoming more conscientious.

However, to date, there are no specific evidence-based interventions focused on increasing conscientiousness (Javaras et al., 2019) and the limited research base on improving conscientiousness is mostly focused on clinical settings (e.g., Javaras et al., 2019; Roberts et al., 2017). Therefore, an important avenue for future research is to investigate how conscientiousness can be fostered in higher education, for example in the form of student support services. This also relates to prior research that has shown that an intervention in college focused on dealing with challenges and taking responsibility for one’s behavior improved coping and resilience (Steinhardt & Dolbier, 2008). Further investigating the effectiveness of such interventions and their potential effect on students’ conscientiousness(-related behaviors) is a promising avenue for future research. Moreover, it may prove effective to communicate to new students that conscientiousness-related behaviors (e.g., being organized in their work, maintaining good attendance, meeting deadlines without support from instructors) are expected of them as they have little understanding about necessary college skills and what is expected of them (Rodriquez et al., 2017). Interventions that foster students’ levels of conscientiousness could be especially valuable for male students who generally score lower on conscientiousness. If research succeeds in designing training or guidance to increase (male) students’ conscientiousness, this could not only increase their academic achievement but may also result in other short- and long-term benefits (e.g., higher job performance; e.g., Barrick & Mount, 1991; better health outcomes; e.g., Bogg & Roberts, 2004) both for the individual and the society at large.

This study yielded more insight into why differences in academic achievement between male and female students exist. That is, the results provided support for conscientiousness explaining the gender gap in academic achievement. This can serve as a first step to develop strategies to reduce the magnitude of gender differences which are disadvantageous for male students (Sax & Harper, 2007).

## Data Availability

The data that support the findings of this study are available from the corresponding author upon reasonable request.

## Appendix 1: Establishing Measurement Invariance of Conscientiousness and Achievement by Ethnic Background

Before examining (possible differences by ethnic background in) the mediation model, invariance across groups of the measurement model was established for a two-factor confirmatory factor analysis model with the latent variables conscientiousness and achievement. Due to inadequate model fit of the initial model of configural invariance (no constraints), three correlated residual terms were added to the model for students with a dominant ethnic background based on MIs and theoretical grounds (see Appendix 2 for the added correlated residual terms). This resulted in good model fit. Accordingly, the model for weak (equal factor loadings) and then for strong (both equal factor loadings and intercepts) measurement invariance was specified. Although the chi-square difference test was significant in both cases, there was no decrease in model fit according to the other goodness-of-fit indices, leading us to accept the model of strong measurement invariance. This model showed good fit to the data, χ^2^(81) = 506.21, *p* ≤ .001, RMSEA = .05, CFI = .95, TLI = 0.94, and SRMR = .04. The factor loading of each indicator to its latent factor was high and significant (standardized factor loadings ranged from .46 to .77 and .45 to .83 for students with a dominant and non-dominant ethnic background, respectively, all *p* < .001).

## Appendix 2: Standardized Factor Loadings of the Indicators of Conscientiousness and Achievement by Ethnic Background

**Table:**

	Students with a dominant ethnic background		Students with a non-dominant ethnic background	
	Standardized factor loadings	*R*^2^	Standardized factor loadings	*R*^2^
*Conscientiousness*				
1. Does a thorough job	.53	.29	.60	.37
2 Perseveres until the task is finished	.46	.21	.48	.23
3 Tends to be disorganized^r^	.65	.42	.69	.47
4 Tends to be lazy^r^	.74	.55	.78	.61
5 Is a reliable worker	.47	.22	.46	.21
7 Makes plans and follows through with them	.54	.29	.54	.29
8 Is easily distracted^r^	.57	.33	.61	.37
9 Can be somewhat careless^r^	.64	.40	.63	.40
2 with 5	.26			
2 with 7	.22			
5 with 7	.19			
*Achievement*				
GPA	.89	.80	.97	.94
Credits	.51	.26	.46	.21

Conscientiousness item 6 “Does things efficiently” was removed due to a low corrected item-total correlation and an higher Cronbach’s alpha after removal. Items marked with ‘*r*’ are recoded.

All estimates are significant at *p* < .001.

## Footnotes

1 Note that the terms “gender” and “sex” are often conflated in the literature (Glasser & Smith, 2008). In our literature review and other parts of this article, we deliberately use the term “gender”, which is cultural and is used when referring to women and men as social groups, rather than “sex”, which is used when the biological distinction is predominant (APA, 2012).

2 I.e., countries in Africa, Asia (including Turkey, excluding Japan, Indonesia), and Latin America.

3 All students are required to participate in the matching program, also if they enrolled at another study program at the university in question or another university before. However, for most students (87.6%) it was their first year of enrollment at Utrecht University.

## Code Availability

The code (Mplus syntax) that support the findings of this study are available from the corresponding author upon reasonable request.
